# Detection of Hepatocellular Carcinoma in an Orthotopic Patient-Derived Xenograft with an Epithelial Cell Adhesion Molecule-Specific Peptide

**DOI:** 10.3390/cancers16162818

**Published:** 2024-08-10

**Authors:** Xiaoli Wu, Shuo Feng, Tse-Shao Chang, Ruoliu Zhang, Sangeeta Jaiswal, Eun-Young K. Choi, Yuting Duan, Hui Jiang, Thomas D. Wang

**Affiliations:** 1 Department of Internal Medicine, Division of Gastroenterology, University of Michigan, Ann Arbor, MI 48109, USA; wuxls@zju.edu.cn (X.W.); shuof@umich.edu (S.F.); jaiswals@med.umich.edu (S.J.); 2 Department of Mechanical Engineering, University of Michigan, Ann Arbor, MI 48109, USA; tsechang@umich.edu; 3 Department of Biomedical Engineering, University of Michigan, Ann Arbor, MI 48109, USA; rlzhang@umich.edu; 4 Department of Pathology, University of Michigan, Ann Arbor, MI 48109, USA; ekchoi@med.umich.edu; 5 Department of Biostatistics, University of Michigan, Ann Arbor, MI 48109, USA; yutingd@umich.edu (Y.D.); jianghui@umich.edu (H.J.)

**Keywords:** hepatocellular carcinoma, EpCAM, peptide, imaging, fluorescence, near infrared

## Abstract

**Simple Summary:**

Hepatocellular carcinoma (HCC) is the most common form of liver cancer, and is occurring with greater frequency worldwide. Better methods are needed to detect this disease at an early time point and help guide surgery. A peptide, or protein fragment, has been developed to bind specifically to EpCAM, a molecular target expressed uniquely by cancer cells and to concentrate in tumors. This peptide was labeled with a near-infrared fluorophore, and showed strong binding to HCC cells and in a live animal model using viable human HCC tumors. The peptide was found to be stable in serum, and showed high uptake in HCC tumors with no signs of toxicity. The peptide also bound to cancer that spread throughout the liver and into the lungs, and was more concentrated in tumors than in other organs. These results support the potential usefulness of the EpCAM peptide to detect and remove HCC.

**Abstract:**

Hepatocellular carcinoma (HCC) has emerged as a major contributor to the worldwide cancer burden. Improved methods are needed for early cancer detection and image-guided surgery. Peptides have small dimensions that can overcome delivery challenges to achieve high tumor concentrations and deep penetration. We used phage display methods to biopan against the extra-cellular domain of the purified EpCAM protein, and used IRDye800 as a near-infrared (NIR) fluorophore. The 12-mer sequence HPDMFTRTHSHN was identified, and specific binding to EpCAM was validated with HCC cells in vitro. A binding affinity of k_d_ = 67 nM and onset of k = 0.136 min^−1^ (7.35 min) were determined. Serum stability was measured with a half-life of T_1/2_ = 2.6 h. NIR fluorescence images showed peak uptake in vivo by human HCC patient-derived xenograft (PDX) tumors at 1.5 h post-injection. Also, the peptide was able to bind to foci of local and distant metastases in liver and lung. Peptide biodistribution showed high uptake in tumor versus other organs. No signs of acute toxicity were detected during animal necropsy. Immunofluorescence staining of human liver showed specific binding to HCC compared with cirrhosis, adenoma, and normal specimens.

## 1. Introduction

Liver cancer results in ~906,000 annual deaths globally, and has emerged as a major contributor to the worldwide cancer burden [1]. Hepatocellular carcinoma (HCC) contributes to ~80% of these cancers. Due to the low number of early-stage diagnoses, the 5-year survival rate for HCC is less than 7%, with a median survival time of under one year [2]. In the U.S., the incidence of HCC is increasing rapidly, and is currently outpacing the growth rate of all other cancers [3]. Indeterminate liver nodules (<2 cm) are frequently seen on abdominal imaging. These conventional modalities are sensitive to the anatomic structure of the mass, but cannot distinguish between benign versus malignant lesions with small dimensions [4]. Major liver societies have recommended that these patients enroll in a surveillance program with serial imaging; however, the endpoint is unclear [5]. New imaging methods that are sensitive to the biological properties of these nodules are needed to improve performance for early HCC detection. Also, new tissue targets are needed to improve in vivo imaging methods for early cancer detection, more accurate diagnosis, and image-guided surgery. While some progress has occurred with serological markers, few advances have been made with identification of HCC tissue targets [6].

EpCAM (epithelial cell adhesion molecule) is a promising tissue biomarker for HCC [7,8,9,10,11,12]. Using immunohistochemistry (IHC), up to 77% of HCC specimens have been found to stain positively for EpCAM on the hepatocyte surface [13,14,15,16,17]. Also, dominant expression has been found in small-nodular HCC tumors [18,19]. EpCAM is a type I transmembrane glycoprotein that normally functions as an epithelial-specific intercellular cell-adhesion molecule [20]. EpCAM is a direct transcriptional target gene for Wnt-β-catenin signaling in HCC cells [21], and is considered to be a biomarker for human epithelial tissues and malignant epithelial tumors. EpCAM functions in cell–cell adhesion, and stimulates cell migration, metastasis, proliferation, and differentiation [22]. EpCAM positive HCC cells possess cancer stem cell (CSC) traits, including the capacity for self-renewal, differentiation, tumorigenesis, and chemotherapy resistance. EpCAM expression as a CSC marker is associated with aggressive behavior and poor clinical outcomes by comparison with conventional tumors [23]. Also, EpCAM overexpression is associated with poor HCC differentiation, short overall survival, and reduced disease-free survival [10].

Image-guided surgery is currently being performed using indocyanine green (ICG), a non-specific contrast agent, to improve tumor visualization [24]. Peptides are promising ligands for in vivo use to visualize overexpressed tumor targets, and can be developed to bind cell surface targets with high affinity and fast kinetics [25]. Peptides can achieve selective tumor uptake in vivo within ~1–2 h after intravenous administration (versus >48 h for ICG), and are stable in serum with a half-life of several hours. This time scale is compatible with clinical use. These ligands clear quickly from the circulation, minimize biodistribution to non-target tissues, and generate minimal background. Peptides by comparison with antibodies have much smaller dimensions to better diffuse and extravasate through leaky vessels and achieve higher concentrations and deeper penetration in solid tumors [25,26]. Also, these ligands have lower potential for immunogenicity, and can be used repetitively [27,28]. Peptides can be labeled with IRDye800, a NIR fluorophore with spectral properties similar to that of ICG. Here, we aim to identify a peptide ligand specific for EpCAM and demonstrate specific binding to human HCC for use during image-guided surgery.

## 2. Materials and Methods

### 2.1. Cell Culture and Chemicals

Human Hep3B HCC cells were sourced from American Type Culture Collection (ATCC), and grown in Eagle’s minimum essential medium (EMEM) with 10% heat-inactivated fetal bovine serum (FBS) at 37 °C and 5% CO_2_. All media and reagents came from Gibco, and IRDye800CW maleimide from LI-COR Biosciences. All products were analytical in grade, and were used as received without any modification.

### 2.2. NIR Peptide Specific for EpCAM

#### 2.2.1. Peptide Selection

A phage display library that expressed 12-mer peptides (New England Biolabs, Ipswich, MA, USA, Ph.D.-12) with ~10^9^ unique sequences was used to biopan against the extra-cellular domain (ECD) of purified EpCAM protein (Sino Biological Inc., Beijing, China, #10694-H08H) for 4 rounds [29]. A total of 100, 80, 60, and 40 μg of EpCAM ECDs were used for each successive round of biopanning. Candidate phages with the highest levels of enrichment were selected for further evaluation. Phage capture ELISA was used to assess binding to Hep3B cells. Binding interactions between the candidate peptides and the EpCAM ECD were assessed using a structural model for EpCAM (4MZV) and Hex 8.0.0 protein–ligand docking software [30]. Unlabeled peptides were custom synthesized (Biomatik, Kitchener, Canada). Labeling of the peptides involved combining 2 mg of peptide with 1 mg of IRDye800CW maleimide (LI-COR Biosciences, Lincoln, NE, USA, #929-80050) in 2 mL of coupling buffer (0.1 M sodium phosphate, 0.5 mM TCEP, pH 7.4). The mixture was reacted in nitrogen (N_2_) for 2 h at room temperature (RT). Subsequently, the NIR-labeled peptides were isolated via reverse-phase high-performance liquid chromatography (RP-HPLC), ensuring a purity level exceeding 99%. The mass-to-charge ratios (*m*/*z*) were measured using mass spectrometry (Bruker AutoFlex Speed, Billerica, MA, USA, #ESI-Q-TOF). The peptides were lyophilized for storage at −80 °C.

#### 2.2.2. Spectral Measurements

The peptide absorbance and fluorescence emission spectra were measured using a Thermo Fisher Scientific (Waltham, MA, USA), Nanodrop #2000c and Ocean Insight (Orlando, FL, USA), #USB2000+ spectrophotometer, respectively. Prism 5.0 software (GraphPad Inc., San Diego, CA, USA) was used to plot the spectra.

### 2.3. Validation of Specific Peptide Binding and Peptide Characterization

#### 2.3.1. siRNA Knockdown

Knockdown of EpCAM expression in Hep3B cells was measured by Western blot. A total of 3 EpCAM-targeting siRNAs (Sigma Aldrich, St. Louis, MO, USA, #SASI-Hs01-0021-3583, #SASI-Hs01-0021-3689, and #EHU037851) and control siRNA (Sigma, #SIC001) were transfected into Hep3B cells. Rabbit anti-EpCAM antibody (Abcam, Boston, MA, USA, #ab71916) was used to assess EpCAM expression.

#### 2.3.2. Binding Competition

Unlabeled target and control peptide was added to IRDye800-labeled target peptides to compete for binding to EpCAM. Inverted confocal microscopy (Leica, Wetzlar, Germany, Stellaris 5) was used to acquire the NIR fluorescence images, and intensities were measured with custom MATLAB (Mathworks) software (Matlab version R2023a).

#### 2.3.3. Binding Affinity and Binding Onset

The binding affinity of the target peptide was measured by assessing the apparent association constant, k_d_ [31]. The target peptide, tagged with IRDye800, underwent a serial dilution in PBS to achieve concentrations ranging from 0 to 200 nM. Approximately 10^5^ human Hep3B cells were treated with this peptide at 4 °C for an hour, followed by a cold PBS wash. Flow cytometry was then performed to determine the average fluorescence intensities. The apparent association time constant k was measured by incubating ~10^6^ Hep3B cells with 5 μM of peptide for 0, 2, 5, 10, 15, 20, and 30 min at 4 °C. The cells were washed with cold PBS and then fixed with 4% PFA for 30 min at 4 °C before performing flow cytometry analysis.

#### 2.3.4. Serum Stability

IRDye800-labeled target peptide measuring 30 μM was incubated with mouse serum at time intervals ranging up to 24 h at 37 °C, and was monitored by analytical RP-HPLC. The relative peptide concentrations were determined by measuring the area under the peak (Waters, Breeze 2).

### 2.4. Orthotopic HCC Patient-Derived Xenograft (PDX) Model

Fresh de novo specimens of human HCC tumors were obtained by the Tissue Procurement Core (TPC) at the Rogel Cancer Center immediately following surgical resection. The specimens were rinsed, preserved in ice-cold Eagle’s minimum essential medium (EMEM), and delivered to the laboratory within 2 h. NOD.Cg-*Prkdc*^scid^ *Il2rg*^tm1Wjl^/SzJ (NSG) mice (Jackson Laboratory, Bar Harbor, ME, USA, #005557) were obtained at age 7–12 weeks old, and were housed per guidelines of the Unit for Laboratory Animal Medicine (ULAM). These mice carry mutations in severe combined immune deficiency (*scid*), and have a complete null allele of the IL2 receptor common gamma chain (*IL2rg^null^*) [32,33]. Freshly resected HCC tissues no larger than 2 mm^3^ were implanted subcutaneously in the NSG mice. The tumor masses were then grown to ~1000 mm^3^ in size, at which point, the tumors were either cryopreserved, characterized, or dissected again for reimplantation and propagation in other NSG mice. For orthotopic implantation, a small horizontal incision was made below the sternum. The exposed liver was incised with a sharp scalpel horizontally. A subcutaneous tumor fragment measuring about 1 mm^3^ in dimension was inserted into the surgical defect. The area was then closed using an absorbable hemostatic product (Johnson & Johnson, New Brunswick, NJ, USA, surgical). Following successful hemostasis, the liver was repositioned into the original location. Orthotopic HCC mice were used for imaging ~3–4 weeks post-implantation.

### 2.5. In Vivo Whole-Body Imaging

IRDye800-labeled target and control peptides (300 μM, 200 μL PBS), unlabeled target peptide (block, 1.5 mM, 100 μL), and ICG (2.46 mg/kg) were injected in tumor-bearing animals using the tail vein. Unlabeled target peptide was injected 30 min prior to the IRDye800-labeled target peptide to compete for binding. The Pearl Trilogy (LI-COR Biosciences) was used to collect whole-body NIR fluorescence images over a time frame of 24 h post-injection.

### 2.6. Laparoscopic Imaging

PDX-implanted mouse livers were exposed by making a small (1–2 cm) incision in the abdomen below the sternum. A custom laparoscope that collects white light and fluorescence images (λ_ex_ = 785 nm) concurrently were used to image the liver region at 1.5 h post-injection via the tail vein [34]. IRDye800-labeled peptides, unlabeled peptide, and ICG were administrated as described above. Once imaging was completed, major organs, including the heart, spleen, lung, liver, brain, stomach, kidney, and intestine, were collected and imaged with white light and fluorescence to evaluate peptide biodistribution.

After the completion of imaging, a human-specific anti-cytokeratin antibody (CAM 5.2, BD Biosciences, Franklin Lakes, NJ, USA, #345779) that reacts to human cytokeratin 7 and 8 was used to validate the presence of patient-derived tissues within the mouse liver.

### 2.7. Animal Necropsy

Healthy mice were euthanized at 48 h post-injection with the IRDye800-labeled target peptide (300 μM, 200 μL PBS). Major organs, including liver, kidney, heart, lung, spleen, stomach, intestine, and brain, were resected and submitted for pathological evaluation to assess for acute toxicity.

### 2.8. Ex Vivo Peptide Validation with Human Liver Specimens

Formalin-fixed, paraffin-embedded (FFPE) sections from *n* = 78 human liver specimens, including *n* = 36, 25, 5, and 12 of HCC, cirrhosis, adenoma, and normal, respectively, were obtained from the archived tissue bank in the Department of Pathology. These specimens were assembled in a tissue microarray (TMA) to enable uniform staining. EpCAM expression was detected with a 1:200 dilution of recombinant anti-EpCAM antibody (Abcam, #ab71916). Confocal microscopy (Leica, Stellaris 5) was used to collect fluorescence images. The mean signal level from 3 boxes (20 × 20 μm^2^ in dimensions) selected at random from each image was measured using custom MATLAB version R2023a.

## 3. Results

### 3.1. NIR Peptide Specific for EpCAM

**Figure 1:**
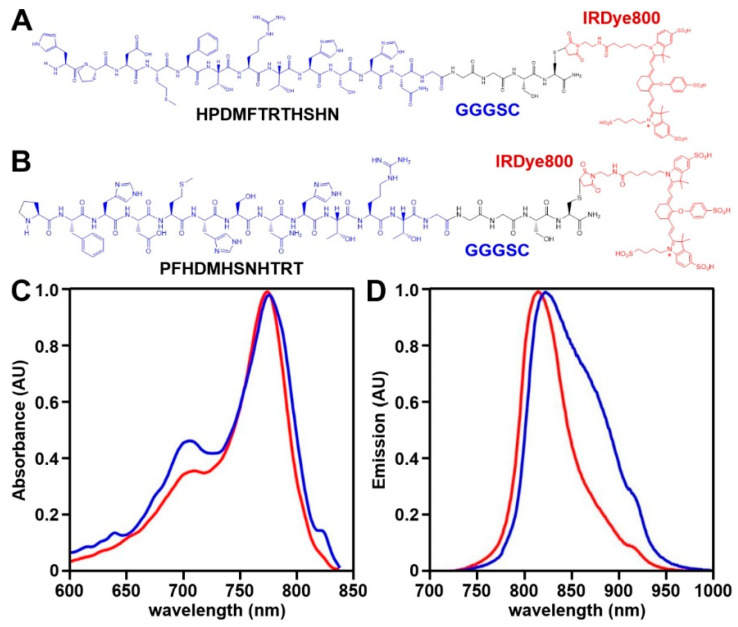
EpCAM-specific peptide. (**A**) Twelve amino acid sequences HPDMFTRTHSHN (target, blue) and (**B**) PFHDMHSNHTRT (scrambled control, blue) were labeled with IRDye800 (red) via a GGGSC linker (black), hereafter HPD*-IRDye800 and PFH*-IRDye800. Peak (**C**) absorbance (red) and (**D**) fluorescence emission (blue) wavelengths were identified at λ_abs_ = 776 nm and λ_em_ = 816 nm, respectively.

A 12-mer phage display library was biopanned against the purified extra-cellular domain (ECD) of the EpCAM target protein. The sequence HPDMFTRTHSHN, hereafter HPD*, showed the greatest enrichment after four rounds of biopanning and the highest absorbance on phage capture ELISA, respectively (Figure S1A,B). IRDye800 was attached covalently to the C-terminus of this peptide using a GGGSC linker, hereafter HPD*-IRDye800 (Figure 1A). Steric hindrance was prevented by separating the peptide and IRDye800 with this linker. The target sequence was scrambled as PFHDMHSNHTRT for control, and was also labeled with IRDye800 via a GGGSC linker, hereafter PFH*-IRDye800 (Figure 1B). The control sequence was identified using a structural model for EpCAM (PDB:4MZV) (Figure S1C). A purity level >95% was achieved for the synthesized peptides by HPLC. Mass spectrometry was used to measure a mass-to-charge ratio (m/z) of 2966.05 (Figure S2A,B). This experimental value was in agreement with the theoretical value of 2966.05. The absorbance and emission peaks of the peptides were observed in the NIR spectrum (Figure 1C,D). In this region, hemoglobin absorption, tissue scattering, and tissue autofluorescence are minimal.

### 3.2. Specific Peptide Binding

#### 3.2.1. Knockdown of siRNA

**Figure 2:**
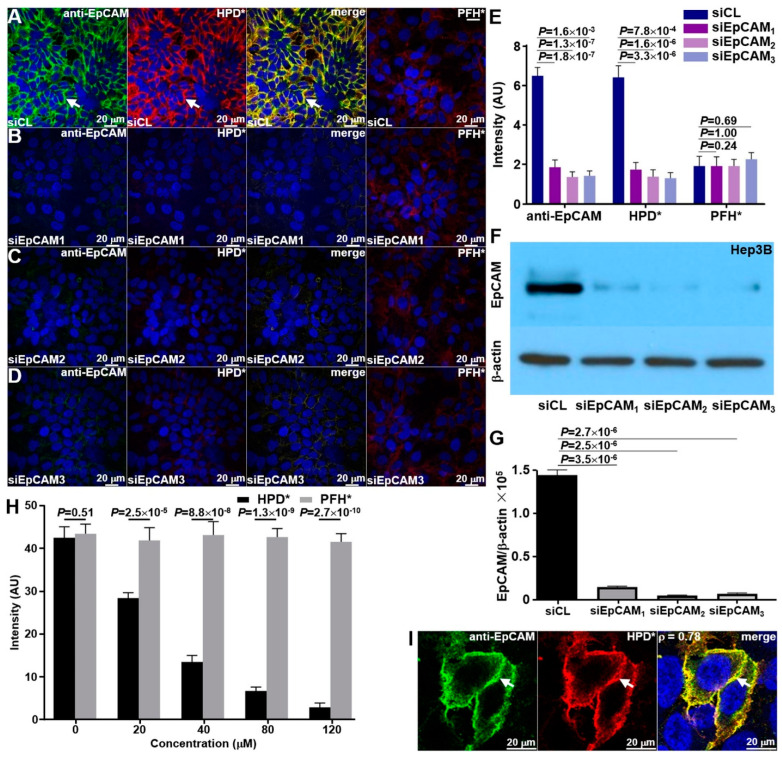
Validation of peptide specificity. (**A**) Anti-EpCAM-AF488 (green) and HPD*-IRDye800 (red) exhibited strong surface binding (indicated by arrows) on Hep3B cells transfected with control siRNA (siCL). The merged image clearly shows co-localization for binding. In contrast, PFH*-IRDye800 (scrambled control) displayed minimal binding. (**B**–**D**) Fluorescence intensities measured for antibody and peptide were greatly reduced with EpCAM knockdown using 3 unique siRNAs. (**E**) Quantified fluorescence intensities showed a significant difference for anti-EpCAM-AF488 with 2.57-, 4.78-, and 4.58-fold changes and for HPD*-IRDye800 with 2.71-, 4.65-, and 4.94-fold changes relative to siEpCAM_1_, siEpCAM_2_, and siEpCAM_3_, respectively. PFH*-IRDye800 showed no significant differences with 1.07-, 1.00-, and 0.85-fold changes. (**F**) Western blot showed EpCAM expression for control (siCL) and knockdown (siEpCAM_1_, siEpCAM_2_, siEpCAM_3_) of Hep3B cells. Results are representative of 5 independent experiments for each measurement. The uncropped blots are shown in Figure S7. (**G**) Quantified Western blot intensities are shown. (**H**) Binding by HPD*-IRDye800 to Hep3B cells decreased significantly with competition from unlabeled HPD* but not with the addition of unlabeled PFH* (scrambled control). Quantified fluorescence intensities showed a concentration-dependent reduction and significantly lower intensity when unlabeled HPD* was added relative to an equal concentration of PFH*, resulting in 0.98-, 0.68-, 0.32-, 0.17-, and 0.08-fold changes, respectively. (**I**) HPD*-IRDye800 and anti-EpCAM-AF488 bind to the surface (arrows) of Hep3B cells with a Pearson correlation coefficient of ρ = 0.78, R^2^ = 0.99, measured on the merged image. All *p*-values were calculated using a two-sample *t*-test.

Specific binding of HPD*-IRDye800 to EpCAM was validated in vitro by performing siRNA knockdown experiments using cultured Hep3B human HCC cells. Using confocal microscopy, anti-EpCAM-AF488 antibody and HPD*-IRDye800 showed strong fluorescence intensity at the surface (arrows) of Hep3B cells transfected with the siCL (control) siRNA (Figure 2A). Minimal binding was seen with PFH*-IRDye800. The fluorescence intensities measured were substantially reduced in the Hep3B knockdown cells for all three unique siRNAs (Figure 2B–D). The decrease was found to be significant (Figure 2E). An effective reduction in EpCAM in the Hep3B knockdown cells was supported by Western blot (Figure 2F). Intensity ratios for each band are shown in Figure 2G.

#### 3.2.2. Binding Competition

The unlabeled HPD* (target) peptide was added to Hep3B cells to compete with HPD*-IRDye800 for binding to EpCAM. Fluorescence intensities were found to decrease significantly with increasing concentrations of unlabeled HPD*. The intensity decrease was found to be concentration-dependent (Figure 2H). However, the addition of unlabeled PFH* (control) did not show an intensity reduction. The results support mediation of binding interactions by the target peptide rather than by either the linker or fluorophore.

#### 3.2.3. Binding Co-Localization

Co-localization of HPD*-IRDye800 and anti-EpCAM-AF488 binding to the surface (arrows) of Hep3B cells showed a correlation of ρ = 0.78 measured on the merged image (Figure 2I).

### 3.3. Peptide Characterization

#### 3.3.1. Binding Affinity and Onset

**Figure 3:**
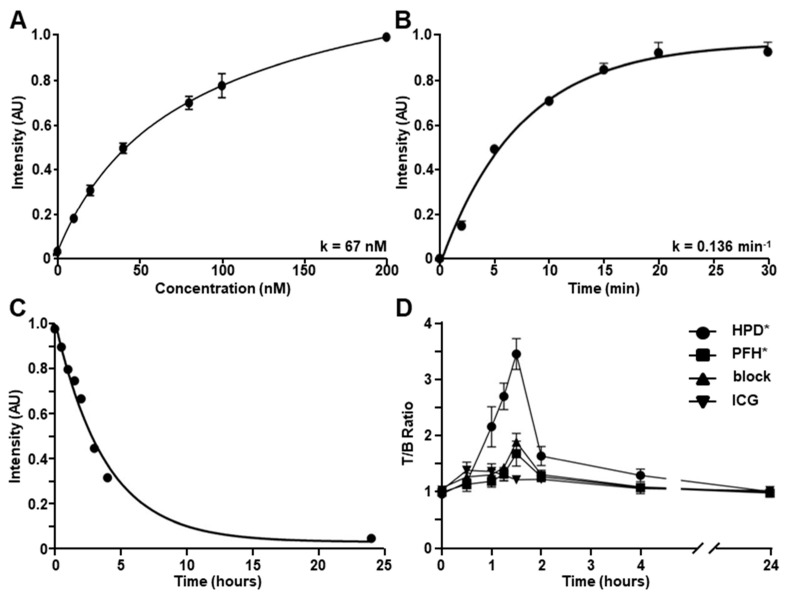
Characterization of peptide parameters. (**A**) An apparent association constant of k_d_ = 67 nM, R^2^ = 0.99, and (**B**) an apparent association of time constant k = 0.136 min^−1^ (7.35 min), R^2^ = 0.98, was measured for binding of HPD*-IRDye800 to human Hep3B HCC cells. Results are representative of 3 independent experiments for each measurement. (**C**) A half-life of T_1/2_ = 2.6 h in mouse serum, R^2^ = 0.99, was measured for HPD*-IRDye800. A total of *n* = 6 mice in each group was evaluated. (**D**) Peak uptake of HPD*-IRDye800 by HCC tumor on whole-body imaging in vivo occurred at 1.5 h post-injection. The quantified T/B ratio for HPD*-IRDye800 was significantly greater than that for PFH*-IRDye800, unlabeled HPD* (block), and ICG with mean ± SD values of 3.45 ± 0.28, 1.67 ± 0.23, 1.92 ± 0.06, and 1.26 ± 0.05, respectively. A total of *n* = 6 animals in each group were evaluated.

An apparent association constant of k_d_ = 67 nM was measured for binding by HPD*-IRDye800 to Hep3B cells to support strong affinity (Figure 3A). An apparent association time constant of k = 0.136 min^−1^ (7.35 min) was measured. This result supports a rapid onset for binding (Figure 3B).

#### 3.3.2. Serum Stability

HPD*-IRDye800 was incubated with mouse serum for time intervals up to 24 h to assess serum stability. The solution was assessed using analytical RP-HPLC (Figure S3). The area under the peak was used to determine the relative concentration. A half-life of T_1/2_ = 2.6 h was measured (Figure 3C).

### 3.4. In Vivo Whole-Body Imaging

**Figure 4:**
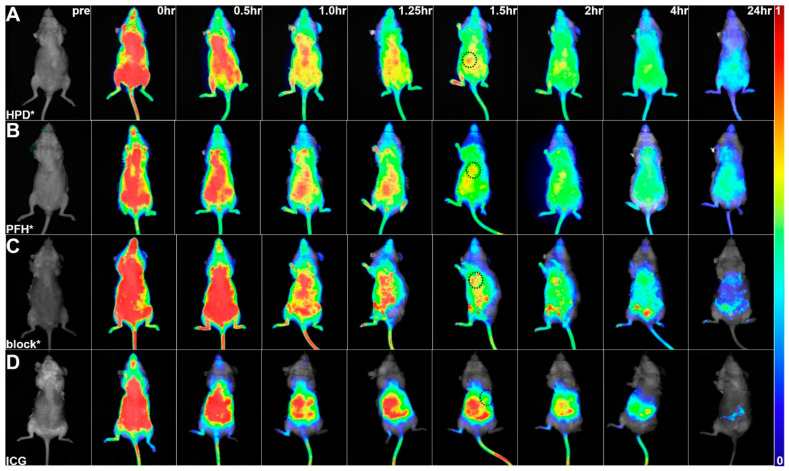
In vivo whole-body fluorescence images. (**A**) Images were collected with excitation at λ_ex_ = 800 nm before (pre) and at 0, 0.5, 1.0, 1.25, 1.5, 2, 4, and 24 h post-injection of HPD*-IRDye800. The peak T/B ratio from the tumor site (circle) was observed at 1.5 h. (**B**) PFH*-IRDye800 was administered for use as control. (**C**) Unlabeled HPD* was injected 20 min prior to HPD*-IRDye800 to compete for binding (block). These controls showed reduced values over 24 h. (**D**) ICG (control) showed non-specific uptake. Representative images are shown from an orthotopic PDX model of HCC with *n* = 6 animals per group.

Prior to the injection of the IRDye800-labeled peptide (pre), all tumors showed negligible fluorescence intensity (Figure 4A). The intensity of HPD*-IRDye800 peaked at 1.5 h post-injection on whole-body fluorescence images, and returned to baseline by ~24 h. The control peptide PFH*-IRDye800 also peaked at 1.5 h post-injection, and showed a reduced T/B ratio at tumor regions as compared with HPD*- IRDye800 (Figure 4B). Unlabeled HPD* (7 mM, 200 μL) was injected 20 min prior to HPD*-IRDye800 to compete for binding to EpCAM (block), and showed a similar pattern with control peptide (Figure 4C). ICG was also administered as a control, and showed strong background in the liver (Figure 4D). Quantified intensities confirmed that the mean T/B ratio for HPD*-IRDye800 was found to be significantly greater than that for PFH*-IRDye800, block, and ICG at peak uptake (Figure 3D).

### 3.5. Laparoscopy

#### 3.5.1. Detection of Primary Tumor

**Figure 5:**
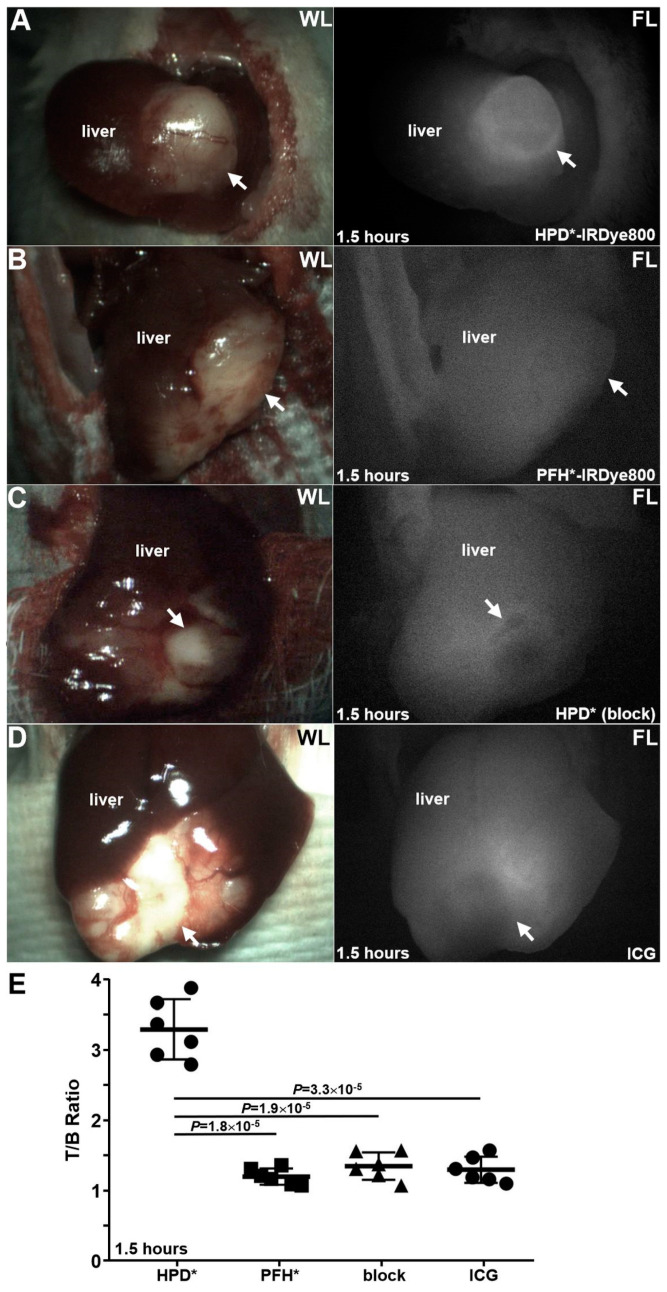
In vivo NIR fluorescence laparoscopic imaging. Representative white light (WL) and fluorescence (FL) images of mouse liver were collected laparoscopically in vivo at 1.5 h post-injection of (**A**) HPD*-IRDye800, (**B**) PFH*-IRDye800 (control), (**C**) HPD* (block), and (**D**) ICG, tumors were designated with arrows. An orthotopic PDX model of HCC was used with *n* = 6 animals per group. (**E**) Quantified NIR fluorescence intensities showed that the mean (±SD) T/B ratio for HPD*-IRDye800 was significantly greater than that for PFH*-IRDye800, HPD*, and ICG with 2.71-, 2.41-, and 2.50-fold increases, respectively. All *p*-values were calculated using a two-sample *t*-test.

HPD*-IRDye800, PFH*-IRDye800, unlabeled HPD* (block), and ICG were administered intravenously in the experimental animals. A standard surgical laparoscope was connected to a custom imaging system to simultaneously capture both white light (WL) and NIR fluorescence (FL) images. Representative white light and NIR fluorescence images are shown in Figure 5A–D. The mean T/B ratio from the tumor was measured at 1.5 h post-injection. The results for HPD*-IRDye800 (target) were significantly greater than that for PFH* -IRDye800 (control), HPD* (block), and ICG, respectively (Figure 5E).

#### 3.5.2. Detection of Local Micrometastases

**Figure 6:**
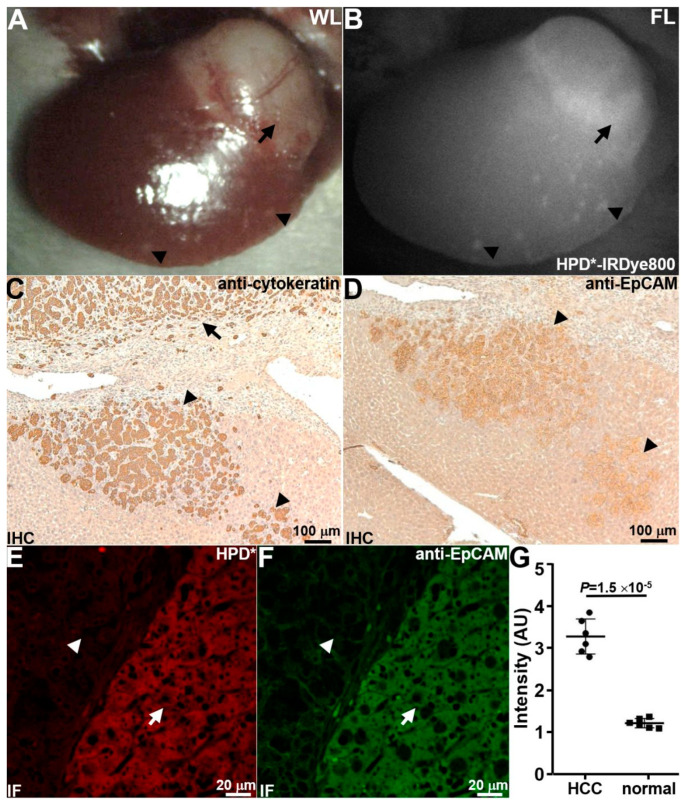
Liver micrometastases detected. Representative (**A**) white light (WL) and (**B**) fluorescence (FL) images of mouse liver collected laparoscopically in vivo at 1.5 h post-injection of HPD*-IRDye800 are shown. In addition to the primary human HCC PDX tumor (arrow), the foci of metastases (arrowheads) were identified. (**C**) Human-specific anti-cytokeratin stain showed the presence of primary HCC PDX tumor (arrow) and the nearby foci of metastases (arrowheads). (**D**) Anti-EpCAM stain confirmed target expression in metastatic foci (arrowheads). (**E**) HPD*-IRDye800 (red) and (**F**) anti-EpCAM-AF488 (green) showed strong binding to HCC (arrow) using immunofluorescence (IF). (**G**) Quantified fluorescence intensities were significantly greater for HCC versus normal with mean ± SD of 3.30 ± 0.42 versus 1.21 ± 0.17, *p* = 1.5 × 10^−5^ by a two-sample *t*-test.

In addition to the primary human HCC PDX tumors (arrow), the foci of micrometastases (arrowheads) seen under white light (WL) (Figure 6A) were also visualized on NIR fluorescence (FL) laparoscopy (Figure 6B). The mice were euthanized after the completion of imaging, and the liver was resected. An anti-cytokeratin and anti-EpCAM stain were performed on these tissue foci to confirm human origin and verify target expression by metastatic tumors (arrowheads), respectively (Figure 6C,D). HPD*-IRDye800 (red) and anti-EpCAM-AF488 (green) showed strong staining for human HCC (arrow) versus adjacent mouse liver (arrowhead) to further verify the orthotopic location of human tumor (Figure 6E,F). The mean fluorescence intensity for HPD*-IRDye800 was found to be significantly greater for tumor versus adjacent normal liver (Figure 6G).

Also, HPD*-IRDye800 was able to identify local sites of HCC micrometastasis to liver on microscopic examination. A group of EpCAM+ cells (arrows) showed strong staining with HPD*-IRDye800 (cyan) and anti-EpCAM (green) (Figure S4A,B). HCC foci < 50 mm in dimensions were identified. The DAPI and merged images are shown in Figure S4C,D. The anti-cytokeratin stain confirmed cells with human origin (Figure S4E). Histology (H&E) confirms the presence of HCC (Figure S4F).

#### 3.5.3. Detection of Distant Micrometastases

HPD*-IRDye800 was able to identify distant sites of HCC micrometastasis in lung on microscopic examination. A cluster of EpCAM+ cells (arrows) showed strong staining with HPD*-IRDye800 (cyan) and anti-EpCAM (green) (Figure S4G,H). The DAPI and merged images are shown in Figure S4I,J. The anti-cytokeratin stain confirmed cells with human origin (Figure S4K). Histology (H&E) supported the presence of tumor in lung (Figure S4L).

### 3.6. Peptide Biodistribution

Major organs from mice-bearing HCC PDX tumors were harvested at 1.5 h post-injection of HPD*-IRDye800, PFH*-IRDye800, HPD* (block), and ICG. NIR fluorescence images were collected, and the intensities were quantified (Figure S5A–D). Tumor uptake of HPD*-IRDye800 was found to be significantly higher than that of the other classifications (Figure S5E). High uptake was observed in kidney, where the peptide was cleared.

### 3.7. Animal Necropsy

Vital organs were collected from normal healthy mice at 48 h following systemic administration of HPD*-IRDye800. No signs of acute peptide toxicity were found on histopathology in major organs, including the heart, liver, spleen, lung, kidney, stomach, intestine, and brain (Figure S6A–H).

### 3.8. Ex Vivo Peptide Validation with Human Liver Specimens

**Figure 7:**
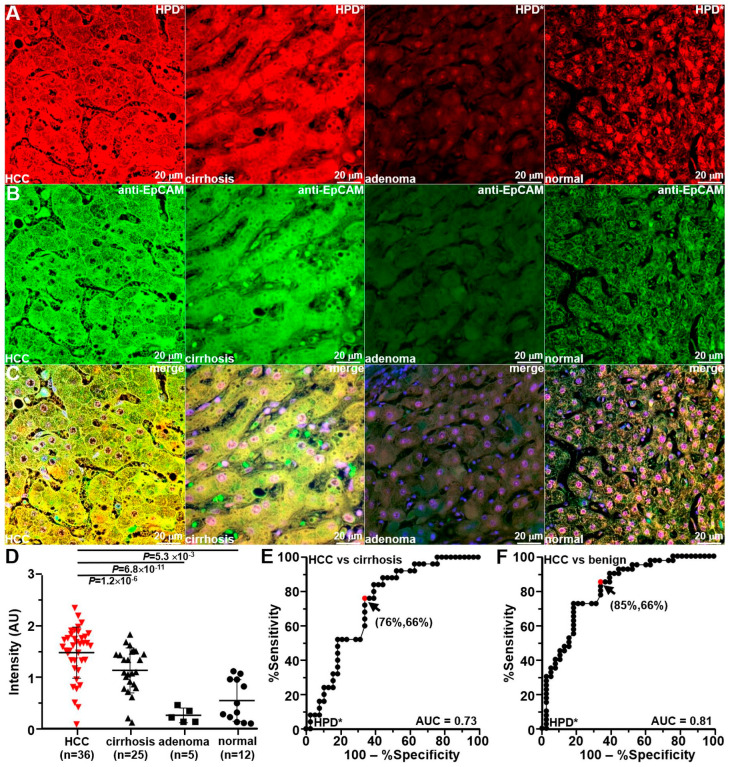
Specific binding to human liver. (**A**) HPD*-IRDye800 (red) and (**B**) anti-EpCAM-AF488 (green) showed intense binding to HCC. Dispersed signal was detected in cirrhosis, while adenoma and normal liver showed only mild staining. (**C**) Merged images showed a Pearson correlation coefficient of ρ = 0.82, 0.79, 0.70, and 0.71, respectively. (**D**) Quantified fluorescence intensities for HCC were significantly greater versus adenoma, cirrhosis, and normal with mean ± SD values of 48.9 ± 16.5, 37.5 ± 14.5, 8.4 ± 4.7, and 18.0 ± 13.4, respectively. A total of *n* = 78 human specimens were evaluated. *p*-values were calculated using a two-sample *t*-test. (**E**) The ROC curve indicates that HPD*-IRDye800 has a sensitivity of 76% and a specificity of 66% for distinguishing HCC from cirrhosis, with an AUC of 0.73. (**F**) Additionally, the ROC curve shows a sensitivity of 85% and a specificity of 66% for distinguishing HCC from benign liver specimens, including cirrhosis, adenoma, and normal tissue, with an AUC of 0.81.

Immunofluorescence staining of formalin-fixed, paraffin-embedded (FFPE) specimens of human liver, including HCC, cirrhosis, adenoma, and normal, was performed using HPD*-IRDye800 (red) and anti-EpCAM-AF488 (green). The specimens were arranged in a tissue microarray (TMA) consisting of *n* = 78 specimens from different patients to evaluate the specific binding of the target peptide and EPCAM antibody to human HCC (Figure 7A,B). The peptide and antibody showed strong intensity with HCC, while moderate diffuse staining was observed for cirrhosis. Adenoma and normal human liver showed minimal staining. Strong co-localization of peptide and antibody binding was seen on the merged fluorescence images with each histological classification (Figure 7C). An expert liver pathologist (EKC) performed the histopathological evaluations. The mean (±SD) fluorescence intensity was significantly greater for HCC by comparison with that for the other histological classifications following staining with HPD*-IRDye800 (Figure 7D). A sensitivity of 76%, specificity of 66%, and AUC = 0.73 was found on the ROC curve to distinguish HCC from cirrhosis (Figure 7E). A sensitivity of 85%, specificity of 66%, and AUC = 0.81 was found from the curve to distinguish HCC from non-tumorous liver (cirrhosis, adenoma, and normal) (Figure 7F).

## 4. Discussion

We have identified a 12-mer peptide HPDMFTRTHSHN that binds specifically to EpCAM. This cell surface target is highly overexpressed in human HCC versus non-tumor [7,8,9,10,11,12] and is a promising tissue target. The peptide was NIR-labeled with IRDye800 to demonstrate usefulness for fluorescence-guided surgery. The validation of specific binding was demonstrated by knockdown, competition, and co-localization experiments using human HCC cells. The peptide was characterized by a binding affinity of k_d_ = 67 nM and an onset of k = 0.136 min^−1^ (7.35 min). Serum stability was measured with a half-life of T_1/2_ = 2.6 h. Patient-derived xenograft (PDX) tumors were implanted in the liver of immunocompromised mice. HCC PDX tumors demonstrated peak peptide uptake in vivo at 1.5 h following intravenous injection. Specific uptake of the IRDye800-labeled target peptide was demonstrated by whole-body fluorescence imaging and NIR laparoscopy in vivo. The mean fluorescence intensity for the peptide was significantly greater versus controls and ICG (non-specific). In addition to the primary tumor, the foci of metastases could be visualized with NIR fluorescence imaging that could not be seen with white light. The peptide was also able to identify micrometastases in the liver and lung. Animal necropsies revealed no signs of toxicity. Staining of human HCC sections demonstrated a high degree of accuracy in differentiating HCC from cirrhosis.

Other ligands specific for EpCAM have been demonstrated in pre-clinical models. An IRDye800-labeled chimeric monoclonal antibody 323/A3 was used to detect a broad range of tumors found clinically [35]. Cell-derived xenograft colon, breast, head and neck, and peritoneal carcinomatosis tumors were implanted orthotopically. Tumor nodules with millimeter dimensions were detected with NIR fluorescence imaging that were not otherwise visible. Peak tumor uptake was observed at 72 h post-injection. Also, a 7-mer peptide YQ-S2 was labeled with the NIR fluorophore MPA [36]. The peptide was characterized by a binding affinity of k_d_ = 118 nM and an onset of k = 0.11 min^−1^ (9.09 min). Cell-derived xenograft breast tumors were implanted orthotopically, and metastatic lesions were detected in the lung and lymph nodes. Furthermore, this peptide was labeled with ^99^Tc for use as a SPECT tracer. A 7-mer peptide SNFYMPL was used to label a pH-sensitive polymeric micelle that encapsulated paclitaxel [37]. This peptide was characterized by a lower binding affinity of k_d_ = 164 nM. Cell-derived xenograft tumors for colon and gastric cancer were implanted subcutaneously, and systemic therapy showed inhibited growth in vivo.

In this study, an orthotopic tumor model was used to validate the specific uptake of the NIR-labeled peptides by HCC for in vivo imaging. This location provides an intact tumor microenvironment that allows for EpCAM expressing HCC epithelial cells to engage in cell–cell and cell–stromal interactions. The implanted patient-derived tissues demonstrated metastatic behavior by spontaneously seeding adjacent liver and distant migration to lung. Also, this location provides representative vasculature to deliver the NIR-labeled peptides. Patient specimens were used to mimic the complex multistep process and tumor–host interactions involved in liver carcinogenesis. These tumors provide clinically relevant target expression levels and molecular heterogeneity. Chemically induced, genetically engineered, and viral HCC tumor models do not accurately recapitulate the HCC tumor biology and tissue targets found in a broad HCC patient population [38,39]. Previously, subcutaneous tumor xenografts were widely used; however, these models have fallen out of favor because of their lack of adequate tumor microenvironment. In the orthotopic location, tumor volume cannot be measured with calipers, but dimensions can be monitored non-invasively using whole-body fluorescence imaging to determine the optimal timing for laparoscopic imaging.

Image-guided surgery aims to remove all cancerous tissues, and thus requires an effective strategy to discriminate tumor cells from non-tumor cells. NIR fluorescence imaging can be used as an adjunct to conventional laparoscopy to assist the surgeon to identify small and missed lesions. The spectra of white light illumination and IRDye800 fluorescence are sufficiently separated so that both modalities can be used concurrently during surgery. ICG is FDA-approved, and this contrast agent is currently being used clinically to identify HCC in real time [40]. The non-specific NIR dye gathers in tumors through the enhanced permeability and retention (EPR) effect [41]. In contrast to HPD*-IRDye800, which peaks at 1.5 h, ICG reaches maximum tumor absorption after 48 h post-injection. Moreover, HPD*-IRDye800 showed sharp tumor margins versus ICG. The EPR effect is largely determined by the molecular weight of the contrast agent. Molecules ranging in size from 40 to 800 kDa have been shown to exhibit an EPR effect [42]. Our peptide has a much lower molecular weight, thus non-specific retention due to EPR will clear quickly. Tumor foci (<200 mm) were visualized via NIR fluorescence imaging with laparoscopy that could not be seen with white light. Additional validation of specific peptide uptake by human HCC tumors will be needed for the future clinical translation of HPD*-IRDye800. Previously, IRDye800 was used to label a 14-mer peptide targeting the gastrin-releasing peptide receptor (GRPR), which was safely utilized in image-guided surgeries of glioblastoma multiforme in *n* = 29 patients [43]. This NIR-labeled peptide is expected to be safe for intravenous use in humans.

In the future, the clinical utility of this EpCAM peptide can be broadened with several improvements. The half-life of T_1/2_ = 2.6 h can be extended by increasing serum stability in a number of ways [44]. Non-proteinogenic amino acids can be incorporated into the sequence to resist protease activity, the N- or C- terminus of the peptide can be blocked with N-acetylation and C-amidation, and the sequence can be arranged in a cyclic structure. Specificity can be improved by repeat biopanning using the phage display library with a further decrease in the quantity of purified protein. The binding affinity can be increased by arranging the peptide monomer in a multimer structure to produce multivalent ligand–target interactions [45]. HCC may be detected at lower levels of expression and at an earlier time point. Limitations of the pre-clinical model used include HCC tumors implanted in a normal mouse liver that did not reflect the presence of chronic hepatitis, fibrosis, or cirrhosis. Also, these tumors took a longer time to grow, and required the use of highly immunocompromised animals. In summary, a peptide that selectively attaches to EpCAM has been identified and validated. The binding properties demonstrate hold significant potential for future clinical use for early cancer detection and image-guided surgery.

## 5. Conclusions

This study highlights potential for the use of specific peptide ligands to detect HCC early. A 12-mer peptide sequence was demonstrated to bind specifically to EpCAM, a tissue biomarker for HCC. This peptide, labeled with IRDye800, demonstrated strong binding affinity and stability, both in vitro and in vivo. The peptide showed high uptake in HCC tumors and metastases, with minimal toxicity and significant specificity compared to other pathology classifications. These findings suggest that this peptide could be a valuable tool to improve early cancer detection and image-guided surgery, ultimately contributing to better clinical outcomes for HCC patients.

## Data Availability

All relevant data are available on request from the authors.

## Supplementary Materials

The following supporting information can be downloaded at: https://www.mdpi.com/article/10.3390/cancers16162818/s1, Figure S1: Phage selection; Figure S2: Mass spectrometry; Figure S3: Serum stability; Figure S4: Validation of micrometastases; Figure S5: Peptide biodistribution; Figure S6: Animal necropsy; Figure S7: Uncropped Western blots.

## Abbreviations

CSC—cancer stem cell; ECD—extra-cellular domain; EpCAM—epithelial cell adhesion molecule; FFPE—formalin-fixed, paraffin-embedded; FOV—field-of-view; HCC—hepatocellular carcinoma; H&E—hematoxylin and eosin; HPLC—high-performance liquid chromatography; IACUC—Institutional Animal Care and Use Committee; ICG—indocyanine green; IHC—immunohistochemistry; NIR—near infrared; NSG—NOD.Cg-*Prkdc*^scid^ *Il2rg*^tm1Wjl^/SzJ; PDX—patient-derived xenograft; ROI—regions of interest; RP-HPLC—reverse-phase high-performance liquid chromatography; RT—room temperature; T/B—target-to-background ratio; TMA—tissue microarray; TPC—Tissue Procurement Core; ULAM—Unit for Laboratory Animal Medicine.
