# Supplementary material for: Detection of Hepatocellular Carcinoma in an Orthotopic Patient-Derived Xenograft with an Epithelial Cell Adhesion Molecule-Specific Peptide
Source: Cancers (Basel). 2024 Aug 10;16(16):2818. doi: 10.3390/cancers16162818 (PMC11352241; doi:10.3390/cancers16162818)
Supplement: Supplementary file 1 [file cancers-16-02818-s001.zip › cancers-2985211-supplementary.pdf]

Supplementary Figures

A

Round	2nd (58 clones)	3rd (59 clones)	4th (59 clones)
HPDMFTRTHSHN	9	9	11
HGLHSMHNKLQD	6	5	6
HPFLHWYNGQRT	3	4	7
DELWMHMHNLTP	2	3	4
NTKKNMIMMKLM	1	12	23

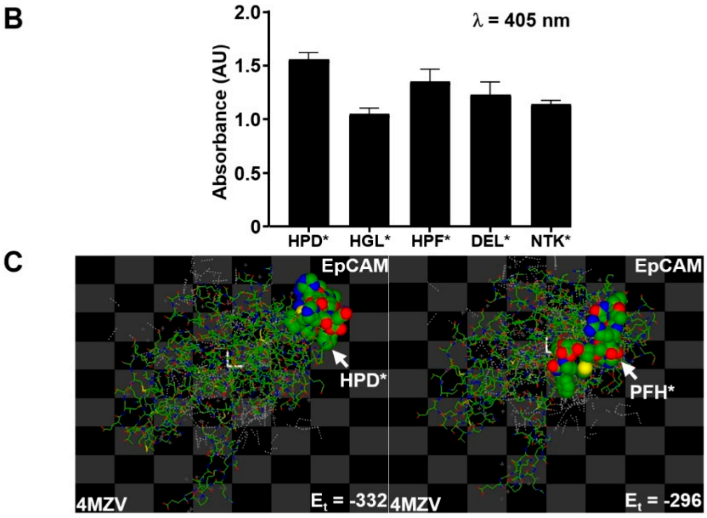

Figure S1 – Phage selection. **A**) Unique clones are shown from phage binding to purified EpCAM protein in successive rounds. **B**) Optical density at 405 nm is shown for candidate sequences using phage capture ELISA assay. Results are representative of 3 independent experiments for each measurement. **C**) The target sequence HPDMFTRTHSHN (HPD*) was scrambled as PFHDMHSNHTRT (PFH*) by evaluating the potential energy E_t using a structural model for EpCAM (PDB:4MZV) with Hex 8.0.0 protein-ligand docking software.

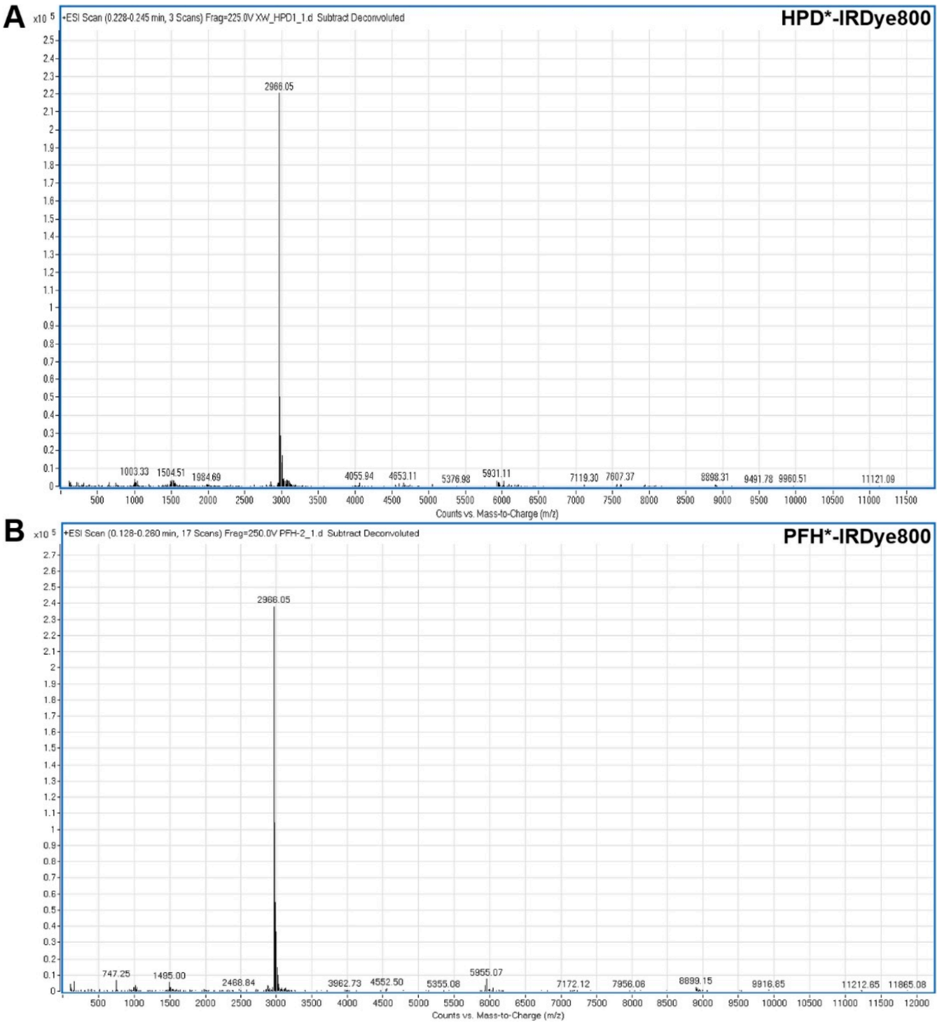

Figure S2 – Mass spectrometry. The experimental mass-to-charge ratios (m/z) for **A**) HPD*-IRDye800 and **B**) PFH*-IRDye800 were found to be 2966.05, which agreed with the expected value of 2966.05.

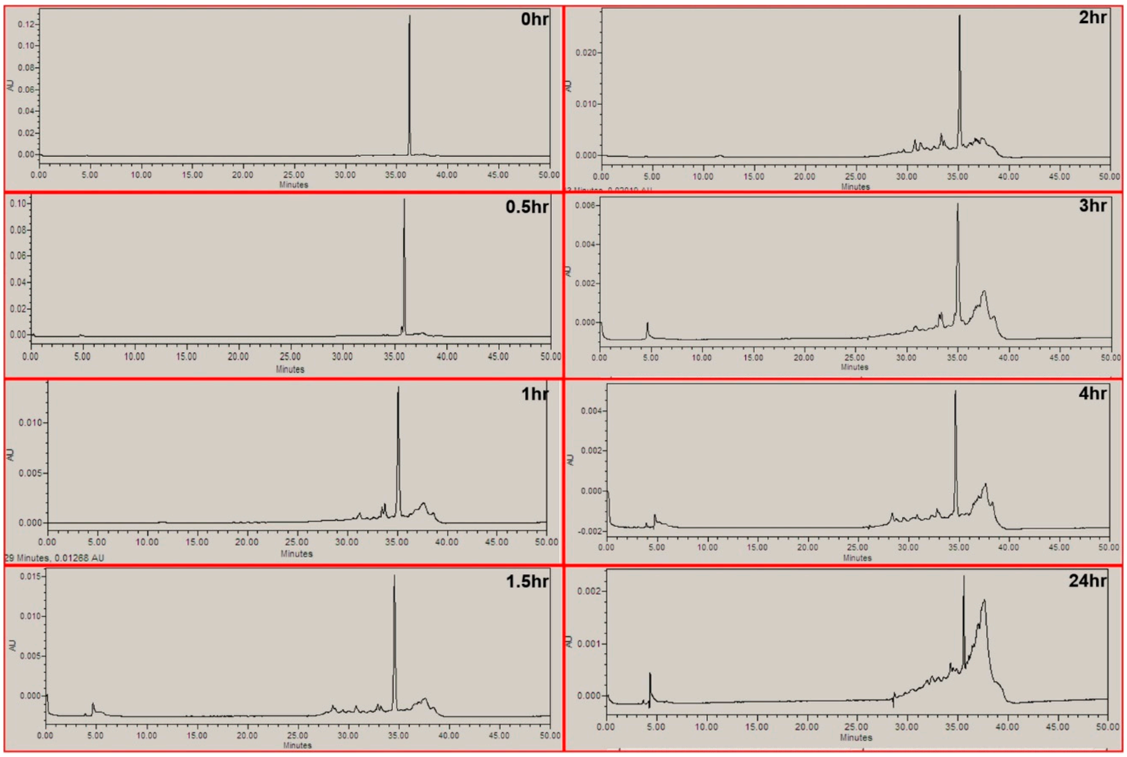

Figure S3 – Serum stability. HPD*-IRDye800 was incubated in mouse serum for 0, 0.5, 1.0, 1.5, 2, 3, 4, and 24 hours. Serum stability was measured using analytical RP-HPLC. The relative concentration was determined by the area-under-the-peak. A half-life of $T_{1/2}$ = 2.6 hours was measured, R^2 = 0.99.

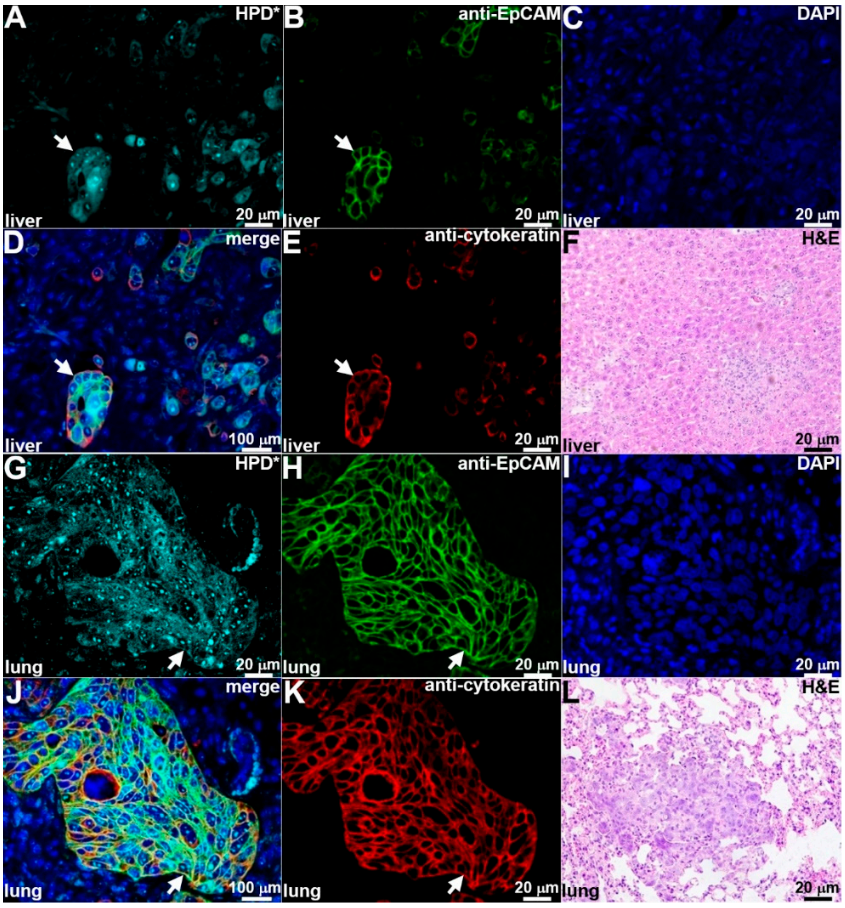

Figure S4 – Validation of micrometastases. Liver: **A**) HPD*-IRDye800 (cyan) and **B**) anti-EpCAM-AF488 (green) showed strong binding to foci of human HCC micrometastases (arrow) in mouse liver. **C**) DAPI stain shows nuclei. **D**) Merged image shows co-binding of peptide and antibody with a Pearson correlation coefficient of $\rho = 0.69$, $R^2 = 0.97$. **E**) Anti-cytokeratin stain confirms presence of human liver tumor (arrow). **F**) Corresponding histology (H&E) verifies human HCC tumor in mouse liver. Lung: **G**) HPD*-IRDye800 (cyan) and **H**) anti-EpCAM-AF488 (green) showed strong binding to foci of human HCC micrometastases (arrow) in mouse lung. **I**) DAPI stain shows nuclei. **J**) Merged image shows co-binding of peptide and antibody with a Pearson correlation coefficient of $\rho = 0.66$, $R^2 = 0.98$. **K**) Anti-cytokeratin stain confirms presence of human tumor (arrow). **L**) Corresponding histology (H&E) verifies human HCC tumor in mouse lung.

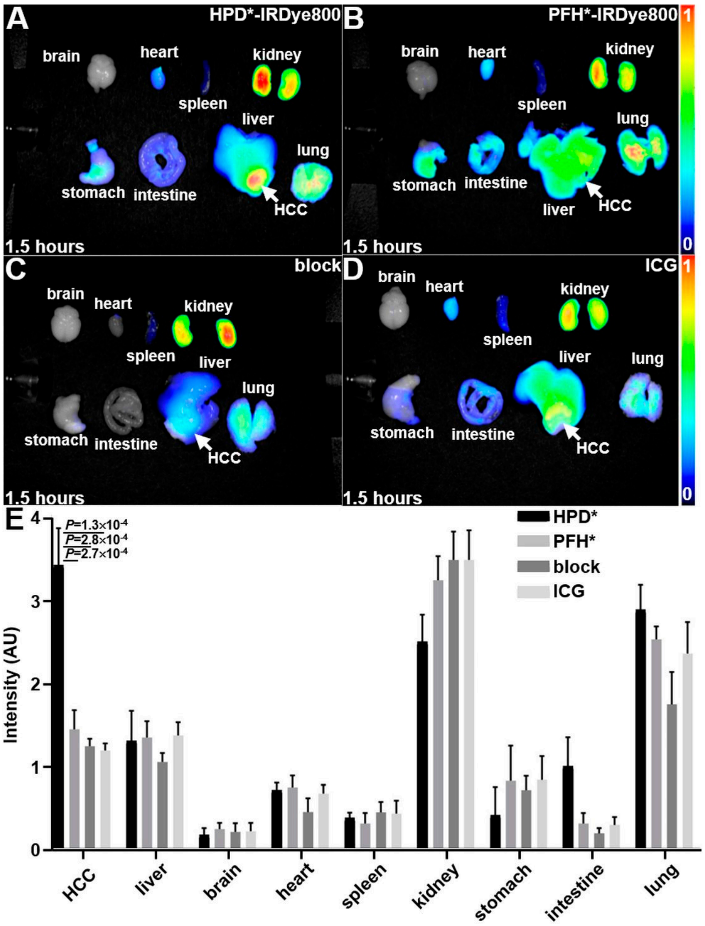

Figure S5 – **Peptide biodistribution**. NIR fluorescence images are shown as an overlay on white light images from major organs at 1.5 hour post-injection of **A**) HPD*-IRDye800, **B**) PFH*-IRDye800, **C**) HPD* (block), and **D**) ICG. **E**) Quantified results showed uptake of HPD*-IRDye800 by the PDX HCC tumor was significantly higher than that for the other groups (3.43 versus 1.46, 1.26 and 1.21 AU). *P*-values were calculated using a two-sample t-test.

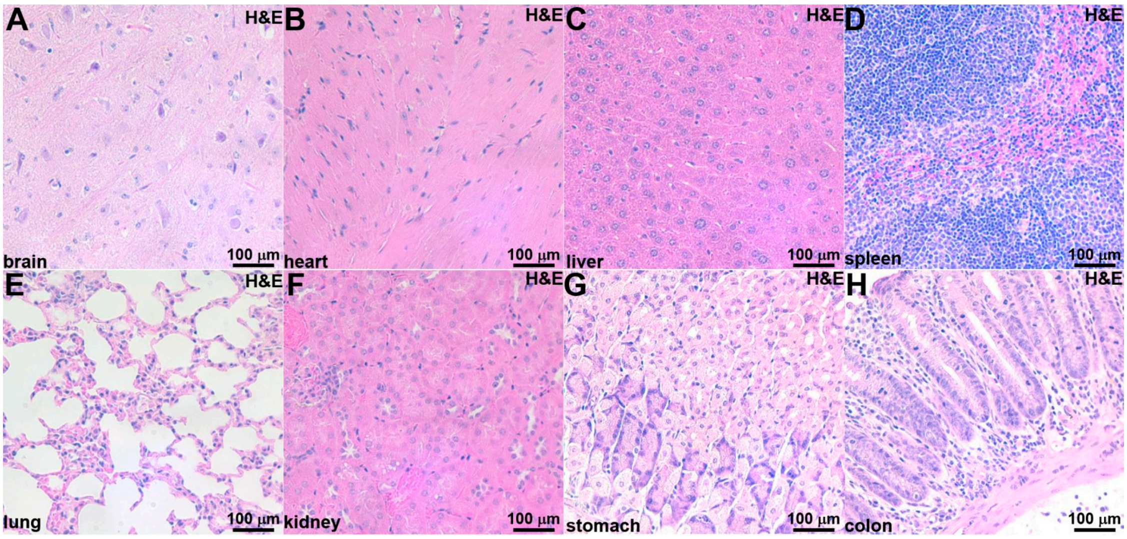

Figure S6 – **Animal necropsy**. Healthy mice were sacrificed at 48 hours post-injection with HPD*-IRDye800 (300µM, 200 µL). No signs of acute toxicity are seen on histology (H&E) of vital organs, including **A**) brain, **B**) heart, **C**) liver, **D**) spleen, **E**) lung, **F**) kidney, **G**) stomach, and **H**) colon.

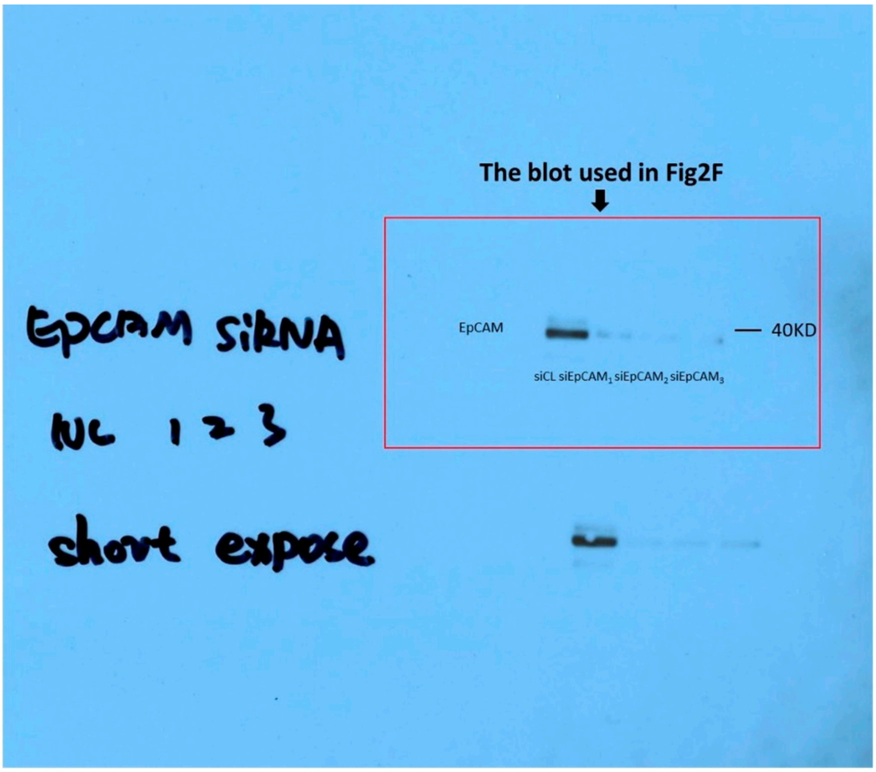

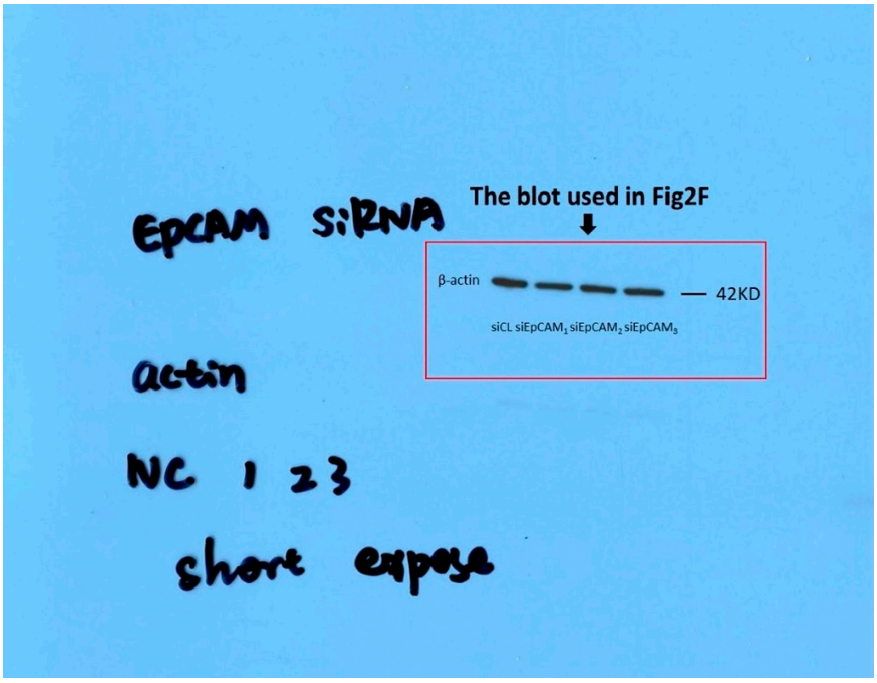

Figure S7 – **Uncropped Western blots**. The original whole blot (uncropped blots) used in Fig2F are shown.
